# COMMBINI: an experimentally-informed COmputational Model of Macrophage dynamics in the Bone INjury Immunoresponse

**DOI:** 10.3389/fimmu.2023.1231329

**Published:** 2023-11-08

**Authors:** Edoardo Borgiani, Gabriele Nasello, Liesbeth Ory, Tim Herpelinck, Lisanne Groeneveldt, Christian H. Bucher, Katharina Schmidt-Bleek, Liesbet Geris

**Affiliations:** ^1^ Biomechanics Research Unit, GIGA-In Silico Medicine, University of Liège, Liège, Belgium; ^2^ Prometheus, Division of Skeletal Tissue Engineering, KU Leuven, Leuven, Belgium; ^3^ Division of Biomechanics, Department of Mechanical Engineering, KU Leuven, Leuven, Belgium; ^4^ Skeletal Biology and Engineering Research Center, KU Leuven, Leuven, Belgium; ^5^ Department of Cell Biology, Erasmus University Medical Center, Rotterdam, Netherlands; ^6^ Julius Wolff Institute, Berlin Institute of Health, Charitè – Universitätsmedizin Berlin, Berlin, Germany

**Keywords:** bone fracture healing, inflammatory phase, macrophages, *in silico* model, multiscale model, sensitivity analysis, genetic algorithm, immunofluorescence

## Abstract

Bone fracture healing is a well-orchestrated but complex process that involves numerous regulations at different scales. This complexity becomes particularly evident during the inflammatory stage, as immune cells invade the healing region and trigger a cascade of signals to promote a favorable regenerative environment. Thus, the emergence of criticalities during this stage might hinder the rest of the process. Therefore, the investigation of the many interactions that regulate the inflammation has a primary importance on the exploration of the overall healing progression. In this context, an *in silico* model named COMMBINI (COmputational Model of Macrophage dynamics in the Bone INjury Immunoresponse) has been developed to investigate the mechano-biological interactions during the early inflammatory stage at the tissue, cellular and molecular levels. An agent-based model is employed to simulate the behavior of immune cells, inflammatory cytokines and fracture debris as well as their reciprocal multiscale biological interactions during the development of the early inflammation (up to 5 days post-injury). The strength of the computational approach is the capacity of the *in silico* model to simulate the overall healing process by taking into account the numerous hidden events that contribute to its success. To calibrate the model, we present an *in silico* immunofluorescence method that enables a direct comparison at the cellular level between the model output and experimental immunofluorescent images. The combination of sensitivity analysis and a Genetic Algorithm allows dynamic cooperation between these techniques, enabling faster identification of the most accurate parameter values, reducing the disparity between computer simulation and histological data. The sensitivity analysis showed a higher sensibility of the computer model to the macrophage recruitment ratio during the early inflammation and to proliferation in the late stage. Furthermore, the Genetic Algorithm highlighted an underestimation of macrophage proliferation by *in vitro* experiments. Further experiments were conducted using another externally fixated murine model, providing an independent validation dataset. The validated COMMBINI platform serves as a novel tool to deepen the understanding of the intricacies of the early bone regeneration phases. COMMBINI aims to contribute to designing novel treatment strategies in both the biological and mechanical domains.

## 1 Introduction

Fracture healing in long bones is a complex process where numerous biological factors cooperate for the complete restoration of the original bone structure and functionality. What makes this process fascinating is the innate capacity of the bone to autonomously initiate its own healing following an injury [Bigham-Sadegh and Oryan (1)]. Immediately after the injury, biological and mechanical factors within the healing region guide the progression of fracture repair [AI-Aql et al. (2); Hankenson et al. (3); Bahney et al. (4)]. The haematoma that forms within the bone fracture has a strong osteoinductive potential [Tsunoda et al. (5); Kolar et al. (6)], generating the environment for successful initiation of the healing process. The early stage of bone fracture healing is characterized by a cascade of events that involves numerous cells, molecules and chemicals recruited from disrupted blood vessels, bone marrow and periosteum niches.

The inflammatory stage is the initial step of bone fracture healing [Schmidt-Bleek et al. (7)]. It starts immediately after the injury as a first response and clears the fracture region of debris, apoptotic cells and necrotic tissue [Niu et al. (8)]. When an open fracture occurs, the inflammatory response prevents the unhindered invasion of external pathogens, thereby reducing the risk of diseases or infection [Loi et al. (9)]. The inflammatory environment is formed promptly after the injury through the invasion and recruitment of specialized cells [Baht et al. (10)], namely innate immune cells. The haematoma region, where the initial phases of healing take place, is formed by a blood clot as a result of disrupted vessels [Kolar et al. (6); Schell et al. (11)] This clot, which contains bone debris and other dead cells forms a region where the inflammatory response is promoted (pro-inflammatory) [Kolar et al. (6)]. The recruitment of innate immune cells such as neutrophils and macrophages will guarantee the cleansing of the healing area from debris and dead cells, which are phagocytized and degraded [Wu et al. (12); Loi et al. (9); Maruyama et al. (13); Gierlikowska et al. (14)]. During the initial inflammation by innate immune cells, a specialized adaptive immune response is triggered with the recruitment and activation of T and B cells, natural killer cells and dendritic cells [Baht et al. (10)]. Especially T cells of the adaptive immune system have been found to regulate the tissue formation beyond the hematoma phase [Reinke et al. (15); Schlundt et al. (16); Bucher et al. (17)]. The innate immune response is initiating the healing cascade whereas the adaptive immune response is dynamically regulating the ongoing inflammatory process. The current version of the COMMBINI model focuses on this inevitable inflammatory stage initiated primarily by macrophages after bone injury.

The physiological development of the inflammatory stage is paramount for the successful repair of the injury [Mountziaris and Mikos (18); Wu et al. (12); Loi et al. (9); Gu et al. (19); Hoff et al. (20); Duda et al. (21)]. However, due to the many factors involved, disruption to the healing cascade is not rare. While some disturbances may have minimal impact, there is a possibility for the occurrence of compromising events, leading to healing delay or non-unions [Bishop et al. (22); Wildemann et al. (23)]. Scenarios where a depleted quantity of macrophages is induced show compromised repair [Alexander et al. (24); Vi et al. (25); Schlundt et al. (26)]. Additionally, prolonged inflammation can have detrimental effects on the healing process, leading to chronic inflammation [Maruyama et al. (13)]. Therefore, it is crucial to regulate and buffer the inflammation (anti-inflammatory response) after a certain number of days [Newman et al. (27)]. Accordingly, a well-coordinated sequence of events is required to generate a suitable environment for the repair and remodeling stages, which will complete the healing process in the following weeks [Baht et al. (10)]. Due to its “dance-opener” role, the successful development of the inflammatory stage is essential to guarantee a productive healing progression. Consequently, many recent studies on bone fracture healing have shifted their focus to this initial stage [Maruyama et al. (13); Newman et al. (27); Baratchart et al. (28)]. Therapeutics and treatments that support the correct initiation of bone fracture healing hold clinical significance in the new generation of biological and mechanical instruments aimed at reducing the risk of failure to heal.

Most of the available literature utilizes *in vitro* models to investigate the immune events that characterize the inflammatory stage of bone healing [Ying et al. (29); Lin et al. (30); Nathan et al. (31)]. However, evaluating the role of dynamics and interactions in the complete scenario remains experimentally challenging. Computer modeling is gaining more and more interest in the academic field for the investigation of mechano-biological processes occurring at multiple levels [Giorgi et al. (32); Vavourakis et al. (33); Lafuente-Gracia et al. (34)]. The possibility to simulate cellular and molecular dynamics and interactions is a valuable asset for the detailed study of bone fracture healing [Borgiani et al. (35); García-Aznar et al. (36)]. Despite their potential, existing computer models of bone fracture healing are mostly limited to the study of the mechano-biological process during repair phases, neglecting the role of the inflammatory stage [Lafuente-Gracia et al. (34)]. To date, only few computer models explored this stage of bone healing by using continuous domains to investigate the dynamics of inflammatory cell and cytokine concentrations [Kojouharov et al. (37); Trejo et al. (38); Baratchart et al. (28)]. However, while those models only evaluate the temporal evolution of the inflammatory cells and cytokines dynamics, the multiscale *in silico* model that we propose employs the computational potentialities to extend the investigation to the spatial dimensions.

In this manuscript, we present a novel *in silico* framework to investigate the mechano-biological interactions in the early inflammatory stage of bone fracture healing at tissue, cellular and molecular levels. A multiscale model is proposed to investigate the interactions between different levels of biological components (*e.g.* cells, cytokines). The agent-based modeling approach provides a new perspective on the role of immune cell populations during the inflammatory stage and their intrinsic capacity to regulate - and be regulated - by the pro- and anti-inflammatory cytokines at the molecular level. The model combines multiple algorithms, to simulate the complete spectrum of multiscale interactions and regulations that happen during the inflammation phase in bone healing. Model calibration was performed using a combination of *in vitro* and *in vivo* results reported in the literature, in part analyzed using a newly developed *in silico* immunofluorescence pipeline. Model validation was executed using an in-house *in vivo* experiment. With this study, we deliver a computational tool that supports the investigation of novel therapeutics and treatments to enhance bone fracture healing with dedicated attention to the multiscale events that interlace during the inflammatory stage.

## 2 Materials and methods

### 2.1 The agent-based model to investigate the cellular level

To investigate the inflammatory stage of bone fracture healing, a multiscale *in silico* model has been developed. The model, named COMMBINI (COmputational Model of Macrophages dynamics in the Bone INjury innate Immunoresponse), aims to simulate the biological and mechanical environment during the progression of the healing of a long bone fracture. To date, only the cellular and molecular modules of COMMBINI have been developed with the support of PhySiCell [Ghaffarizadeh et al. (39)], an open-source software that simulates the cells as single entities within an agent-based model. These virtual cells perform phenotype-specific activities (*e.g.* migration, proliferation) and regulate the molecular level (*e.g.* consumption and production of cytokines).

**Figure 1:**
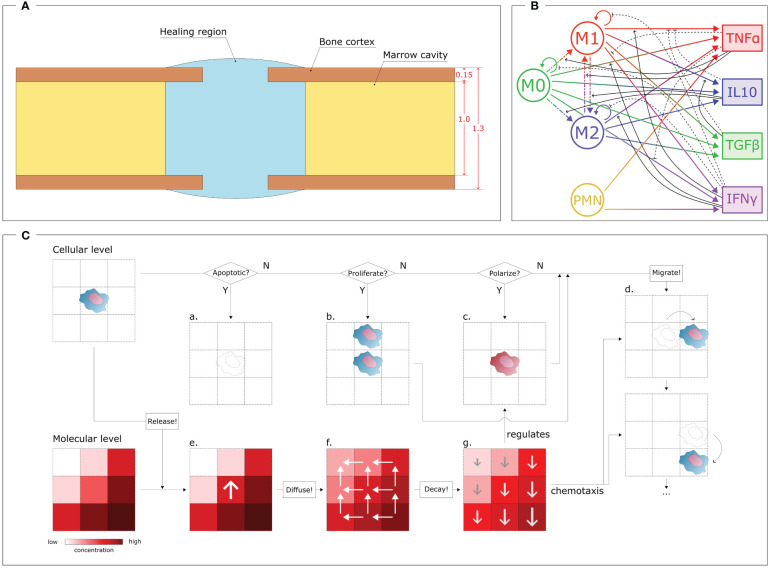
Overview of the COMMBINI components. **(A)** Simulation domain (blue), based on the callus geometry for bone fracture healing, wherein the cellular and molecular levels are simulated. Dimensions reported in mm. **(B)** Multiscale interactions between the cellular level (left) and the molecular level (right). Circular arrows: proliferation/population doubling; dash-dotted arrows: macrophage polarization/interpolarization; gradient arrows: cytokine secretion; black arrows: cellular activity regulations (solid: promotion, dashed: inhibition). M0: non-polarized macrophages, M1: pro-inflammatory macrophages, M2: anti-inflammatory macrophages, PMN: polymorphonuclear neutrophils, TNFα: Tumor Necrosis Factor alpha, IL10: Interleukin 10, TGFβ: Transforming Growth Factor beta, IFNγ: Interferon gamma. **(C)** Schematic representation of the rules that regulate the two levels. At the cellular level (top), for each cell it is checked if the cell is in an apoptotic, proliferative or polarized state, according to dynamics reported in the literature and translated into computer model algorithms. If apoptotic **(a)**, the cell is removed by the model; if proliferative **(b)**, a daughter cell is created in one of the surrounding positions; if polarized **(c)**, the phenotype changes. The cell can migrate **(d)** by performing a sequence of jumps. Each cell releases specific molecules at the molecular level (bottom) by increasing their concentration in the specific position **(e)**. Then, the molecules diffuse **(f)** from regions of high concentrations to low; and degrade **(g)** following exponential dynamics, therefore having a faster decay in more concentrated regions. The molecular environment regulates the polarization algorithm **(c)** and drives cell migration **(d)** through chemotaxis.

During the inflammatory stage, the cellular level plays a major role, as the innate immune cells actively contribute to initiating the healing response. To simulate this cellular level, an agent-based model has been developed. With this approach, each cell was simulated independently and not as a passive component of a cell population, thereby providing stochasticity to the investigation and guaranteeing the spatio-temporal variability that characterizes biological systems [Wehrens et al. (40); Allen et al. (41)]. The simulation was performed within a geometrical domain that represents the shape of a murine tibia fracture over a virtual period of 3 days, encompassing the early inflammatory stage. For the current study, a fracture opening in the center of the bone was simulated. The size of the fracture gap depends on the specific case study under investigation (cfr. § 2.3, 2.7). The model geometry was created by assuming a hollow cylinder as a simplified shape for the bone and a spheroid shape for the callus domain (healing region), following the same assumptions as previous studies [Wang and Yang (42); Borgiani et al. (43); Perier-Metz et al. (44)]. The healing region is the spatial domain where cell activities and molecular dynamics are simulated. Boundary conditions are imposed on the surfaces of the healing region. Bone marrow is simulated as a reservoir of non-polarized macrophages: they are recruited from the bone marrow compartment to invade the healing region. Furthermore, once the inflammation is over, the macrophages leave the region and emigrate back to the marrow compartment. The same conditions are imposed on the curved surface of the healing region, which simulates the periosteal boundaries. A zero-flux condition is imposed on the surface of the bone cortex as it is assumed that cells cannot migrate and cytokines cannot diffuse through it. The 2D model is generated by an intersecting plane along the middle axis (**Figure 1A**).

The iterative nature of the model allowed the investigation of the cellular environment evolution with a time resolution of Δt*_cell_* = 1 min. In each iteration, virtual cells within the Region of Interest (ROI) perform specific actions based on phenotype-specific ratios, and the cellular environment is updated accordingly. Four different cell phenotypes are described in this computer model: non-polarized macrophages (M0), pro-inflammatory macrophages (M1), anti-inflammatory macrophages (M2) and polymorphonuclear neutrophils (PMN) (**Figure 1B**). The PMNs are the only cell type simulated within the healing region at the initial time-point. They are uniformly distributed within the region with an initial concentration [**PMN]_0_**. Macrophages start to appear from the first iterations of the simulation onward. At the molecular level, an initial concentration of fracture debris (**Db_0_**) is homogeneously distributed within the healing region. This initial condition is crucial as the presence of debris chemotactically promotes the invasion of the healing region by the immune cells. No inflammatory cytokines are simulated within the region at the initial time-point but they start being secreted from the first iteration onwards. The M0 recruitment from the marrow cavity and tissues surrounding the healing region is stimulated by the presence of debris. The PMNs and macrophages phagocytose the debris, leading to a decrease in its concentration and recruitment capacity as healing progresses. To simulate this behavior in the computational model, the M0 recruitment ratio follows a dynamic pattern that decreases along with the physiological reduction of debris concentration within the healing region [Trejo et al. (38)]:


(1) $$\frac{\text{ΔM}0}{\text{Δt}}={\text{k}}_{\text{R}(\text{M}0)}(1-\frac{\left[M\Phi \right]}{{\left[\text{M}\Phi \right]}_{\max}})[\text{Debris}]$$

Equation (1) halts macrophage recruitment when no more debris needs to be removed or if the maximal concentration of macrophages is reached within the healing region. The dynamic is regulated by two parameters, whose variation can lead to faster or slower recruitment of macrophages: **k_R(M0)_** is the maximum non-polarized macrophage recruitment ratio and [**MΦ**]**_max_** is the maximal macrophage concentration allowed within the healing region. To create a realistic evolution of the cellular environment during the inflammatory stage of bone healing, the macrophages and PMNs perform additional activities, *i.e.* they migrate, proliferate, polarize and are subject to apoptosis (**Figure 1C**).

In COMMBINI, cellular migration is stochastically simulated as a sequence of “jumps” in random directions to create a movement pathway in the 2D space [Allen et al. (41)]. The span of each jump is defined by the migration speed (**k_v_**) associated with each cell phenotype. Cellular proliferation is simulated by generating a daughter cell with identical characteristics to its mother cell in one of the neighboring positions. The proliferation ratio (**k_p_**) associated with each cell phenotype determines the frequency of cell division within each iteration. Apoptosis is simulated as the removal of cells by programmed cell death. The apoptosis ratio (**k_a_**) of a cell increases with the accumulation of phagocyted debris [Bratton and Henson (45)] and the number of other cells in its vicinity, mimicking the consumption of essential nutrients for survival. Furthermore, macrophages have the ability to change their phenotype in response to the surrounding inflammatory environment as perceived at the molecular environment (more details in § 2.2). The M0 macrophages can, under specific molecular conditions, polarize into either an M1 or M2 phenotype (**Figure 1B**)[Yunna et al. (46)]. In our model, this process is simulated as the change of the phenotype flag associated with the macrophage. Following the phenotype switch, the virtual macrophage adjusts its behavior by modifying its parameter values and algorithm dynamics according to the characteristics assigned to the new phenotype. Moreover, although infrequent, interpolarization can occur between M1 and M2 phenotypes, depending on whether pro-inflammatory macrophages reside in an anti-inflammatory environment, and vice versa (**Figure 1B**)[Yunna et al. (46)]. Inter-polarization into pro-inflammatory macrophages is rare compared to the interpolarization into the anti-inflammatory phenotype due to the natural progression of the bone healing process. To avoid unnecessary complexity, this model does not include further subdivisions within the M2 subtypes. However, the model can be readily expanded to include such subdivisions if the scope is extended beyond the inflammatory stage to incorporate the repair phase.

In the discrete agent-based model, the apoptosis, proliferation and polarization conditions are reported as probability values for the respective event to occur within the iteration period **Δt*_cell_***. Therefore, during each iteration, a random floating point value between 0 and 1 (precision 10^−6^) is assigned to each cell for each event. If the value exceeds the probability value, the event does not get triggered (N paths in **Figure 1C**). Conversely, if the value is lower than the probability value, the cell is removed, generates a daughter cell or changes its phenotype (Y paths in **Figure 1C**). For cell proliferation, the position of the daughter cell is randomly selected from the four adjacent positions that are not occupied by other cells. Migration is performed at every iteration by allowing the cell to jump multiple times to adjacent positions based on their migration speed and the spatial and temporal resolution of the model. In this model, assuming a spatial resolution of 1 µm (cellular model spatial resolution) and an iteration period of Δt*_cell_* = 1 min (temporal resolution), a PMN (k_v_ = 5.00 µm min^−1^) will perform five jumps during each iteration. The direction of each jump is randomly chosen among the four surrounding positions that are not occupied by other cells, when chemotaxis is not involved. However, a large part of the phagocytic cells included in this work is driven by the fracture debris gradient. Chemotaxis is incorporated into the model by directing cell movement according to the gradient of the chemotactic agent concentration (**Figure 1C**).

While macrophages are recruited, PMNs promote the onset of the inflammatory response. In the first version of COMMBINI, PMNs are the only non-macrophage population considered at the cellular level. At the start of the simulation, PMNs are uniformly distributed within the healing region with an initial concentration [PMN]_0_. Through the course of the inflammation, PMNs are recruited from the surrounding tissues by following a dynamic analogous to (1). PMNs are short-lived cells that tend to disappear from the healing region after triggering the initial inflammatory signal and its amplification [Summers et al. (47)]. Therefore, the proliferation of PMNs is not included in the model (**Figure 1B**). To simulate the natural behavior of neutrophils, PMNs simulated in COMMBINI release pro-inflammatory cytokines and clear debris from their surroundings to generate a pro-inflammatory environment [Kovtun et al. (48, 49)].

### 2.2 Differential equations to describe the molecular level dynamics

The cellular level has a mutual regulatory relationship with the molecular level. Consequently, we simulated the molecular model within the same agent-based model that simulates the cellular environment. The dynamics of cytokine concentration at the molecular level are simulated using partial differential equations (PDE) with function descriptions obtained from the literature (**Supplementary Table 2**). The equations were solved using the BioFVM solver [Ghaffarizadeh et al. (50)] on a 2000 µm x 2000 µm square 2D grid within the healing region, with a resolution of 10 µm. The concentration of each inflammatory cytokine is evaluated in each grid element. This setup enables multiscale interactions, as each element in the molecular model shares its position with one or more cells in the cellular environment, according to the common coordinate system. The activities of the cells within the same element are regulated by the cytokine concentration within it (**Figure 1C**). Conversely, the presence of cells within each element regulates the intrinsic variation of cytokine concentration, reproducing phenotype-specific dynamics (**Figure 1C**). Macrophage polarization is regulated by the molecular level as the macrophages simulated at the cellular level polarize according to the cytokine concentration predicted in the same spatial location of the healing region (**Figure 1C**). Tumor Necrosis Factor alpha (TNFα) and Interleukin 10 (IL10) have been chosen for this model to respectively represent pro- and anti-inflammatory cytokines at the molecular level. Therefore, we described the macrophage polarization rules as probability functions, which are regulated by the concentration of those cytokines [Trejo et al. (38)]:


(2) $$\text{P}\left(\text{M}0\to \text{M}1\right)={\text{k}}_{01}\frac{\left[\text{TNF}\alpha \right]}{{\text{a}}_{01}+\left[\text{TNF}\alpha \right]}$$

(3) $$\text{P}\left(\text{M}0\to \text{M}2\right)={\text{k}}_{02}\frac{\left[\text{IL}10\right]}{{\text{a}}_{02}+\left[\text{IL}10\right]}$$

(4) $$\text{P}\left(\text{M}1\to \text{M}2\right)={\text{k}}_{12}\frac{\left[\text{IL}10\right]}{{\text{a}}_{12}+\left[\text{IL}10\right]}$$

(5) $$\text{P}\left(\text{M}2\to \text{M}1\right)={\text{k}}_{21}\frac{\left[\text{TNF}\alpha \right]}{{\text{a}}_{21}+\left[\text{TNF}\alpha \right]}.$$

In equations (2 - 5), the parameters **k** represent the macrophage polarization ratios and the parameters **a** represent the cytokine half-saturation for macrophage polarization.

The molecular environment is, in turn, regulated by the immune cells (**Figure 1B**). These release pro- and anti-inflammatory cytokines, according to the dynamics included in the model. In addition to TNFα and IL10, the model includes Transforming Growth Factor beta (TGFβ) and Interferon gamma (IFNγ), as they regulate cell activity in the healing region: *e.g.* TGFβ lowers secretion of pro-inflammatory cytokines by M1 macrophages [Nagaraja et al. (51)], and IFNγ downregulates macrophage proliferation (**Figure 1B**). All the cell-specific cytokine secretion dynamics simulated in this model are reported in **Table 1**.

**Table 1:** Cell-specific cytokine secretion dynamics for each cytokine included in the *in silico* model.

M0	TNFα	${\text{k}}_{\text{TNF}}(1+\frac{{\text{k}}_{\text{TNI}}}{1+e^{\left({\text{a}}_{\text{TNI}}-[\text{IFN}\gamma ]\right)}})$
	IL10	k_IL10_
	TGFβ	k_TGF_
	IFNγ	k_IFN_ *e* ^−a^_ITN_[TNF*α*]
M1	TNFα	${\text{k}}_{\text{TNF}}\left({\text{k}}_{\text{TNIL}}e^{-{\text{a}}_{\text{TNIL}}[\text{IL}10]}+{\text{b}}_{\text{TNIL}}\right)\left({\text{k}}_{\text{TNTG}}e^{-{\text{a}}_{\text{TNTG}}[\text{TGF}\beta ]}+{\text{b}}_{\text{TNTG}}\right)\left(1+\frac{{\text{k}}_{\text{TNI}}}{1+e^{\left({\text{a}}_{\text{TNI}}-[\text{IFN}\gamma ]\right)}}\right)$
	TGFβ	k_TGF_
	IFNγ	k_IFN_ *e* ^−a^_ITN_[TNF*α*]
M2	IL10	k_IL10_
	TGFβ	k_TGF_
PMN	TNFα	k_TNF_
	IFNγ	k_IFN_

M0: non-polarized macrophages, M1: pro-inflammatory macrophages, M2: anti-inflammatory macrophages, PMN: polymorphonuclear neutrophils, TNFα: Tumor Necrosis Factor alpha, IL10: Interleukin 10, TGFβ: Transforming Growth Factor beta, IFNγ: Interferon gamma.

The cytokines diffuse through the molecular level by following Fick’s law of diffusion, with a specific diffusion coefficient **(D)** associated with each cytokine. Neumann boundary conditions (no-flux) have been assigned to the borders of the healing region and bone cortex. Additionally, decay rates (**d**) have been set for each cytokine to simulate their enzymatic degradation, leading to a decrease in concentration. A temporal resolution of Δt*_mol_* = 1 s was assigned to iteratively simulate the dynamics within the molecular level. To coordinate the temporal dynamics between the two levels, which are characterized by different temporal resolutions, the cellular environment updates every 60 iterations of the molecular level.

Additionally, the molecular level simulates the dynamical spatio-temporal variation of the concentrations of debris within the healing region. In this study, the term debris is used to define the agglomerate of dead cell bodies and necrotic tissue pieces resulting from the bone fracture. The presence of debris elicits the release of Damage Associated Molecule Pattern (DAMP) inflammatory stimuli. The distribution of debris concentration is included at the molecular level as a biological variable capable of influencing the inflammatory stage development [Chow et al. (52)]. In COMMBINI, the macrophages follow the debris concentration gradient at the molecular level to orient their migration towards the zones of the healing region characterized by a higher concentration of debris. Phagocytosis has been implemented in the model as the capacity of macrophages and PMNs to remove debris in their spatial surroundings, hence clearing the healing region. An engulfment ratio **k_e_** was defined to quantify the debris phagocyted by those cells within the iteration period.

### 2.3 Dedicated *in vivo* experiments for model calibration

The model parameters at both cellular and molecular levels were obtained from previously published *in vitro* works that investigated the biological characteristics of macrophages and cytokines (**Supplementary Tables 1, 2**). Afterward, a parameter calibration was performed to minimize the differences between the simulation outcomes and the experimental results from dedicated *in vivo* studies through the use of immunofluorescent imaging of macrophage populations. The *in vivo* experiments have received approval from the Ethical Committee for Animal Experimentation of the KU Leuven (approval number 020/2022). Tibial osteotomies (1 mm) were created in male C57BL/6 mice, fixated with an external Ilizarov fixator as previously described [van Gastel et al. (53)]. Three samples were obtained from the animals at 3 days post-fracture and prepared for immunohistology. The samples were fixated in formalin overnight at 4°C and decalcified with an edetic acid (EDTA) solution. The decalcified fracture samples were embedded in paraffin and 5 µm thick sections were mounted on glass slides. One slide from the center of each sample has been selected for immunofluorescence staining, obtaining *n* = 3 *ex vivo* images to use for calibration. The slides were deparaffinized with Histo-Clear (National Diagnostics, cat. no. HS-202) and dehydrated, followed by enzymatic antigen retrieval using 1 mg mL^−1^ Pepsin in 0.02M HCl. The samples were blocked with 5% bovine serum albumin (BSA) in phosphate-buffered saline (PBS) with 0.1% Tween20 (Merck, cat. no. P1379) and 0.01% Tergitol (AppliChem, cat. no. A9780) for 45 minutes at room temperature. The samples were stained with immunofluorescent markers for macrophages and their specific subtypes. DAPI identifies all the nuclei and the Cluster of Differentiation 68 (CD68) is a general marker for macrophages [Schlundt et al. (26)]. Co-expression of CD68 and CD80 is specific for pro-inflammatory macrophages, while co-expression of CD68 and CD206 identifies anti-inflammatory macrophages [Schlundt et al. (26)]. The samples have been incubated overnight at 4°C with a 1:500 dilution of anti-CD68 antibody (ThermoFisher, cat. no. 14-0681-82) and a 1:100 dilution of anti-CD80 (ThermoFisher, cat. no. PA5-85913) or anti-CD206 antibody (ThermoFisher, cat. no. PA5-101657) in blocking buffer. On the second day, the samples were incubated for 4 hours at room temperature with Goat anti-rat IgG, Alexa Fluor Green 488 antibody with a 1:500 dilution (ThermoFisher, cat. no. A-11006) and Donkey anti-Rabbit IgG, Alexa Fluor Red 594 with a 1:1000 dilution (ThermoFisher, cat. no. A32754) in blocking buffer. Since bone is autofluorescent, the Vector TrueVIEW autofluorescence quenching kit (Vector, cat. no. SP-8400-15) was used. Finally, a counterstaining was performed with 5 µg mL^−1^ DAPI for 10 minutes. The samples were dried and mounted in VECTASHIELD Vibrance Antifade (Vector, cat. no. H-1700). Samples were imaged using the Olympus IX83 inverted microscope within 48 hours. The sections were conserved at -20°C for additional image acquisitions.

### 2.4 Deep-learning cell quantification and *in silico* immunofluorescence

**Figure 2:**
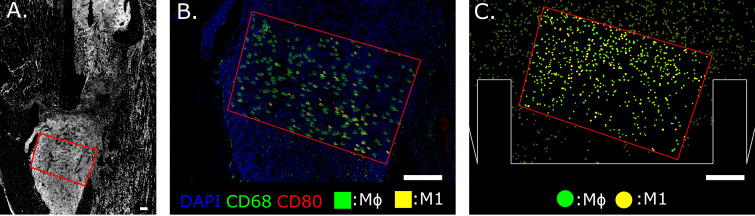
Region of Interest selection in the distal bone fracture **(A)** and cellular level comparison between *ex vivo* **(B)** and *in silico* **(C)** immunofluorescence at day 3 post-fracture. The staining utilized for *ex vivo* immunofluorescence marked nuclei in blue (DAPI), generic macrophages (MΦ) in green (CD68) and pro-inflammatory macrophages (M1) in yellow (co-expression of CD68 and CD80, green + red = yellow). *In silico* immunofluorescence used the same color-code associated with the specific macrophage markers used in the experiments to facilitate a direct qualitative comparison between experimental data and the simulation results. For quantitative comparison, macrophage concentration within the same area (red outline) is compared, located at the callus site indicated in **(A)**. Scalebar = 200 µm.

A custom Python script was developed to analyze the immunofluorescent images and extract quantitative information at the cellular level. The outcome of the pipeline generates a fully segmented image with spatial information about macrophage distribution. Whole-cell segmentation was performed by Mesmer (DeepCell), a deep-learning tool trained on an extensive database of tissue image data and validated by experts [Greenwald et al. (54)]. Dimension filtering is applied to the images and the elements with a surface area below 80 µm^2^ or larger than 200 µm^2^ are not classified as cells [Cannon and Swanson (55)]. An ROI is chosen on the immunofluorescent image by selecting the fracture region, avoiding the bone cortex and staining artifacts. All cells within the ROI are labeled according to phenotype and quantified. For each macrophage phenotype, concentrations are calculated by dividing the number of cells by the ROI area. This data is compared with the macrophage concentrations simulated by the cellular level in the agent-based model. To perform a more direct qualitative comparison, *in silico* immunofluorescence was generated as output of the computational model by assigning the same color-coded pattern to the virtual cells as the immunofluorescent images. For example, the bright green fluorescence assigned to the CD68 channel was used to paint the cytoplasm of all the virtual macrophages, as they are supposed to express that marker (**Figure 2**). Co-marking is represented by the chromatic combination of the two markers: *i.e.* M1 cells that are co-marked by CD68 (green) and CD80 (red) are represented *in silico* with a yellow color (**Figure 2**). Additionally, this novel computational technique delivers a dataframe that contains information about all the cells identified within the ROI of the immunofluorescence image. Each cell is categorized in detail according to its size, the 2D position of its centroid and marker positivity.

### 2.5 Design of experiments to reduce the calibration complexity

The parameter calibration of the computer model was performed by following an optimization pathway to reduce the difference between quantified experimental and simulation outcomes. The calibration process can be time-exhaustive when many model parameters are included. Therefore, a sensitivity analysis was performed to determine the model parameters that most strongly influence the quantitative outcome of COMMBINI. The model was run multiple times with different combinations of parameter values. Reduction of the number of simulation runs was possible by cutting non-necessary repetitions with the support of Taguchi’s orthogonal arrays [Kacker et al. (56)]. This strategy is convenient when many parameters have to be analyzed: the model is regulated by 36 parameters and a 2-level sensitivity analysis would have required 2^36^ simulation repetitions to analyze all the parameter combinations (full factorial). With Taguchi’s orthogonal array, we reduced this number to 72, drastically dropping the estimated runtime of the analysis. An analysis of variance (ANOVA) was performed on the model outputs to evaluate the percentage of the total sum of squares (%TSS) for each parameter [Isaksson et al. (57)]. The absolute value of this percentage represents how sensitive the output is to variation of the parameter value: higher %TSS expresses a more significant influence on the output. The sign associated with the %TSS indicates the influence on the output variation: if positive, an increase in the parameter value results in an increase in the output value and vice versa. For each output, the four most influential parameters were selected according to the highest %TSS absolute value.

### 2.6 Genetic Algorithm to perform the model parameter calibration

Once the most significant parameters were identified by the sensitivity analysis, we calibrated them with the support of a Genetic Algorithm (GA) [McCall (58)]. A fitness function was generated employing data from experimental images, with the aim of reducing the quantitative differences between the *in silico* model and *ex vivo* immunofluorescence images. Numerical differences between experimental data (*e.g.* concentration of macrophages) and the corresponding quantitative output from the agent-based model of the cellular level were employed as the fitness function. The GA follows an evolutionary approach based on subsequent generations, aiming to minimize the fitness function. If a combination of parameter values did not reduce the function, it was removed by the algorithm in the following generation, allowing it to keep only the most promising ones. The selection of the most promising values and their cross-combination with the other components of the population minimized, generation after generation, the fitness function until a predetermined threshold was met. A more detailed explanation of the GA methodology employed to calibrate this model is reported in **Supplementary Materials**.

### 2.7 Model validation with an independent experimental dataset

Validation of the results was performed on a different dataset of experimental immunofluorescent images (*n* = 2), previously reported by Schlundt et al. (26). Differently from the dataset that was used for calibration, the model of the validation set is characterized by a smaller fracture gap size (0.7 mm), in a different bone (femur) from female mice. The mouse strain (C57BL/6) was analogous to our in-house experiment and the same immunofluorescent staining markers were used to investigate the macrophage distribution in *ex vivo* images. The model domain was adapted to match the dimensions of the validation experiment’s bone and fracture gap. The biological parameter values obtained from the GA calibration process were validated by quantitatively comparing the macrophage populations concentrations simulated on this new domain and the ones measured from *ex vivo* immunofluorescent images. The success of the validation process supports the claim that confirms the assertion that the additional calibration step using data from *in vivo* experiments is important and leads to a more accurate representation of the inflammatory phase of fracture healing in murine long bones than when using parameter values derived from *in vitro* experiments reported in the literature.

### 2.8 Statistical analysis of the *in silico* results

Due to the involvement of the discrete agent-based framework, the multiscale model has a stochastic nature. The variability is shown by the mean and standard deviation of multiple repetitions (*n* = 5) of the simulation under the same investigative conditions and different initial random seeds. One-tailed student’s T-test was performed to investigate the differences between the calibrated and non-calibrated models.

## 3 Results

### 3.1 *In silico* immunofluorescence with literature values

**Figure 3:**
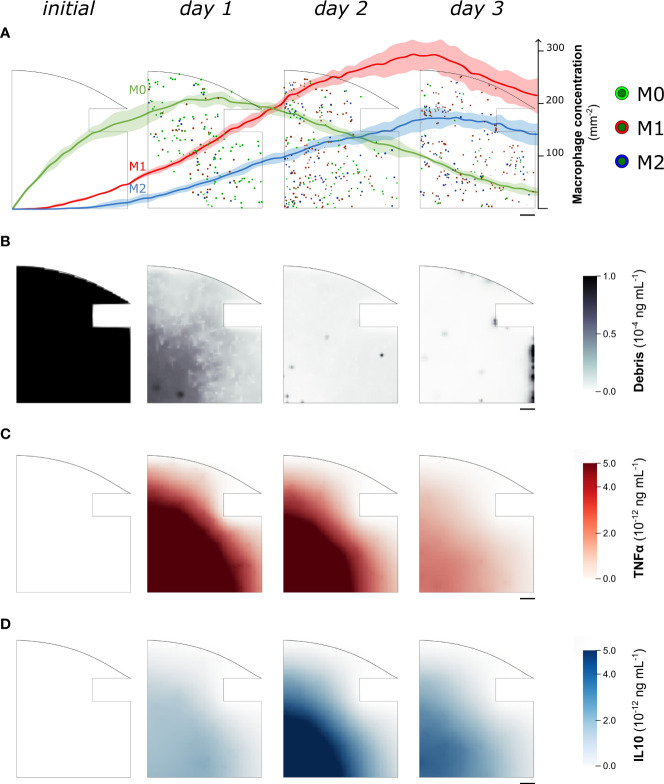
Representative images of the temporal evolution of the cellular level **(A)** and the molecular level **(B–D)** during the fracture healing progression. Model results were collected from one quarter of the healing callus every 24 hours since the fracture induction (initial). In **(A)** we superimposed the quantitative variation of macrophage concentration (mean ± standard deviation, *n* = 5) over the course of the healing process. Neutrophil population is not shown to improve readability. M0: non-polarized macrophages, M1: pro-inflammatory macrophages, M2: anti-inflammatory macrophages, TNFα: Tumor Necrosis Factor alpha, IL10: Interleukin 10. Scalebar = 100 µm.

When *in vitro* experiments reported in the literature are used to parametrize the model, the simulation results show a concentration of macrophages within the healing region of 346.4 ± 9.3 mm^−2^ after 1 day, followed by an average increase of 12.7% between day 1 and day 3. Specifically, at day 1 the M0 concentration is 207.5 ± 8.1 mm^−2^, the M1 concentration is 99.2 ± 7.4 mm^−2^ and the M2 concentration is 39.7 ± 7.1 mm^−2^. As the inflammation progresses, the concentrations vary between day 1 and day 3: M0 decreases by 84.4 ± 4.1%, M1 and M2 increase 2.2-fold (± 0.3) and 3.2-fold (± 0.8) respectively (**Figure 3A**). At the molecular level, the cellular engulfment leads to a reduction in fracture debris over time, resulting in the complete clearance of debris from the healing region within 3 days (**Figure 3B**). Pro- and anti-inflammatory cytokines secreted by immune cells exhibit analogous dynamics throughout the onset of bone healing, though pro-inflammatory cytokine secretion is more intense during the early stage of healing (**Figure 3C**), followed by a delayed anti-inflammatory wave (**Figures 3C, D**).

### 3.2 Sensitivity analysis to evaluate the most influential parameters for *in silico* outputs

When considering the total macrophage concentration output, the ANOVA test revealed that the *in silico* model exhibited the highest sensitivity to the macrophage recruitment ratio (*k_R_*_(_ *_M_*_0)_) during the early stage of inflammation (%TSS = 46.9% at day 1, reduced to %TSS = 3.3% at day 3). In the later stage, it was observed that the initial concentration of PMNs ([PMN]_0_) had the largest impact, although with a negative trend (%TSS = -37.5% at day 3). Additionally, the non-polarized macrophage proliferation ratio (*k_p_*_(_ *_M_*_0)_) influenced the results at day 1 (%TSS = 12.9%), while the pro-inflammatory macrophage proliferation ratio (*k_p_*_(_ *_M_*_1)_) had a greater effect on the output at day 3 (%TSS = 15.9%). Furthermore, the debris engulfment ratio associated with PMNs (*k_e_*_(PMN)_) exhibited an influence on the predicted macrophage concentration at day 3, with a negative trend (%TSS = -13.4%). The complete list of %TSS associated with each parameter at day 1 and 3 is reported in **Supplementary Table 3**.

### 3.3 Genetic Algorithm to identify optimal parameter set

**Figure 4:**
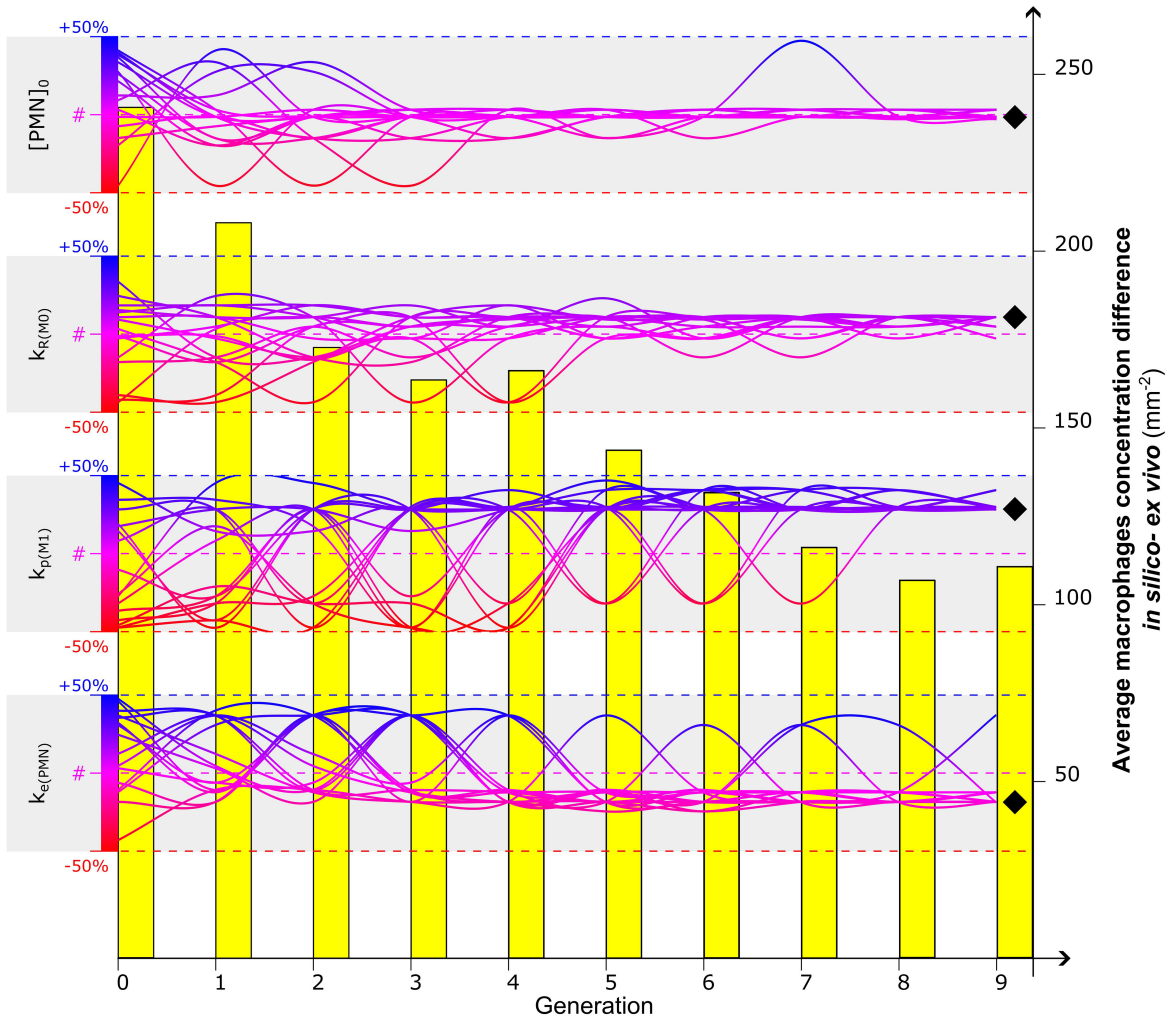
Calibration of the four most influential parameters ([PMN]_0_, k_R(M0)_, k_p(M1)_, k_e(PMN)_) to optimize the *in silico* predicted macrophage concentration at day 3. Evolution of a 16-sample population is represented by the gradient-colored lines: each sample represents a combination of values associated with the four parameters. The Genetic Algorithm is initialized at generation 0 by randomly associating to each parameter a value within the range of +/-50% of the values found in the literature (identified by #) (**Supplementary Tables 1, 2**). The dynamic evolution of the algorithm led the combination of the parameter to converge to values that better calibrate the model. Diverging bumps observed in the evolution of the lines are associated with mutations, singularities of the Genetic Algorithm to increase the investigative variability. After a full run of Genetic Algorithm (9 generations in this case), a value is identified for each parameter to calibrate the model (black diamond). The capacity of the Genetic Algorithm to minimize the fitness function is observed in the evolutionary reduction of the difference of macrophage concentration between *in silico* and *ex vivo* data (yellow bars). For additional details about the Genetic Algorithm, the reader is addressed to **Supplementary Materials**.

By minimizing the fitness function, defined as the difference in the macrophage concentration within the healing region between values obtained from computer simulations and experiments on day 3 postfracture, the GA identified the optimal combination of values for the most influential parameters at that time-point ([PMN]_0_, *k_R_*_(_ *_M_*_0)_, *k_p_*_(_ *_M_*_1)_, *k_e_*_(PMN)_). The algorithm converged after nine generations for the parameters (**Figure 4**), and it resulted in a clear tendency for higher macrophage proliferation rates (*k_p_*_(_ *_M_*_1)_) to better capture the experimental data (1.07 10^−3^ min^−1^, +28.5% compared to literature value). Calibrated values for macrophage recruitment and neutrophil engulfment ratios showed smaller yet still considerable divergence from literature-based values (*k_R_*_(_ *_M_*_0)_ = 2.33 10^−2^ h^−1^, +10.9%; *k_e_*_(PMN)_ = 2.71 10^−3^ min^−1^, -18.6%) and the initial PMN population tended to maintain the concentration value found in the literature ([PMN]_0 _= 984.38 µm^−3^, -1.6%). Throughout the iteration of the GA, the average difference between *in silico* output and *ex vivo* immunofluorescent image quantification decreased from 240.9 mm^−2^ to 107.1 mm^−2^, resulting in a 56.5% reduction of the fitness function (**Figure 4**).

**Figure 5:**
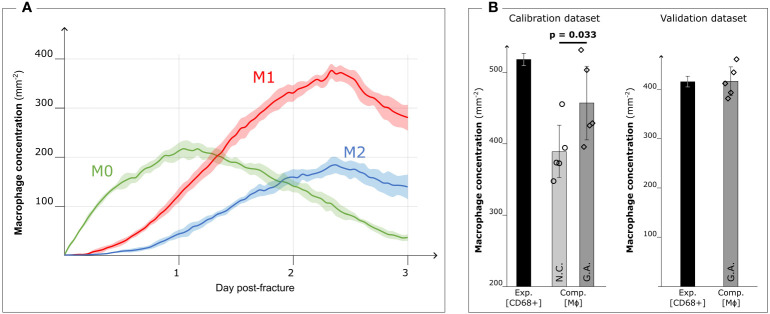
Results after Genetic Algorithm calibration and comparison with experimental data. **(A)** Dynamic variation of the concentration of the different macrophage types (M0: non-polarized macrophages, M1: pro-inflammatory macrophages, M2: anti-inflammatory macrophages, mean ± standard deviation, *n* = 5) over the course of the inflammation progression, when Genetic Algorithm calibrated parameters are used. **(B)** Comparison between the experimental immunofluorescence concentration of macrophages (Exp. [CD68+]) (black bars, mean ± standard deviation, *n* = 3 for calibration dataset, *n* = 2 for validation dataset) and computational predicted concentration of macrophages (Comp. [MΦ]) (gray bars, mean ± standard deviation, *n* = 5) with parameter based on literature data (Non-Calibrated, N.C.) or calibrated with Genetic Algorithm (G.A.). Scatter plots of the 5 results from the computer model are added to show the model stochasticity.

When the model was run with the optimized parameters, the M0 concentration peaked around day 1 (213.1 ± 17.4 mm^−2^) and decreased with the progression of the inflammation (35.7 ± 6.5 mm^−2^ on day 3). Pro- and anti-inflammatory macrophage concentrations increased from day 1 (M1: 122.1 ± 14.6 mm^−2^, M2: 43.2 ± 8.3 mm^−2^) to day 3 (M1: 281.9 ± 26.6 mm^−2^, M2: 140.0 ± 25.4 mm^−2^) (**Figure 5A**). The M1 concentration showed a significant influence of the calibration on day 1 (*p* = 0.016) and day 3 (*p* = 0.008), in contrast to both M0 and M2 concentrations where no significant influence was observed (*p >* 0.05).

When comparing the results of the model using literature-based values (**Figure 3A**) with those obtained with the calibrated model, we observed that the qualitative dynamics of macrophage concentration during the inflammation processes remained unaltered for all the subtypes. However, there was an increase in the number of cells within the healing region. The pro-inflammatory macrophage concentration in particular increased (+31.0%) due to the GA-driven increment of the proliferation ratio. This observation aligns with the fitness objective of the calibration to reduce the difference in macrophage concentration on day 3 between *ex vivo* immunofluorescence (518.8 ± 8.3 mm^−2^, identified as CD68+ cells) and computer model results (non-calibrated: 389.3 ± 36.5 mm^−2^; GA optimized: 457.6 ± 51.5 mm^−2^, *p* = 0.033) (**Figure 5B**).

### 3.4 Validation of the calibration results with an alternative dataset

In the case of the 0.7 mm fracture dataset, we observed a decrease in macrophage concentration within the healing region. The *ex vivo* immunofluorescence data at day 3 showed a concentration of 414.0 ± 22.6 mm^−2^ (CD68+). This reduction in macrophage concentration is also predicted by the GA-calibrated *in silico* model for the 0.7 mm gap. Specifically, the *in silico* model predicted a concentration of 415.2 ± 28.8 mm^−2^ at day 3 post-fracture (**Figure 5B**).

## 4 Discussion

This manuscript presents an integrated *in silico*-*in vivo* pipeline for the development and calibration of a computational model capturing the early phase of fracture healing, called COMMBINI. By employing agent-based modeling, each biological cell is represented as a single discrete entity and not as an element of a dynamic continuous concentration, providing a novel perspective on the investigation of the early phase of the bone healing process. The agent-based model is designed with stochastic algorithms to faithfully reproduce the biological behavior of cells [Andrews et al. (59); Wehrens et al. (40); Allen et al. (41)]. However, COMMBINI also includes deterministic rules to investigate the processes that drive healing progression, such as chemotaxis. These deterministic rules are essential for introducing spatial information and preventing the agent-based model from generating a homogeneous environment. Chemotactic attraction is one of the deterministic factors promoting the directional migration of the immune cells within the healing region [Kolar et al. (6)]. Specifically, debris chemotaxis was observed to be essential to simulate the recruitment of the first macrophages from the bone marrow and surrounding tissues to the center of the fracture gap. The implementation of a spatio-regulated debris clearance rule to reproduce the natural behavior of macrophages [Gordon and Plüddemann (60); Westman et al. (61)] was necessary to complete the callus invasion, reducing the recruitment of further macrophages.

The molecular level has been simulated by solving diffusion-decay differential equations within a region that shares the coordinate system with the cellular level, following the approach in Borgiani et al. (43). The domain size has been chosen to fully include the healing domain and its spatial resolution has been adapted to create a sufficiently fine grid on which to solve the equations, avoiding to increase the computational costs. With the proposed resolution (10 µm), the molecular level is capable of adequately reproducing the cytokine dynamics without increasing the simulation time. Also, temporal resolution differs between the cellular and molecular levels. By following the in-code values proposed by BioFVM, the time resolution has been kept to the order of seconds to guarantee an accurate and smooth simulation of the molecular dynamics, with no detriment to computational performances. These settings have been based on previous benchmarks of the solver, where adequate accuracy has been obtained in diffusion-decay systems under the same temporal resolution utilized in this work [Ghaffarizadeh et al. (50)]. Furthermore, the overall timespan of the inflammatory stage is limited to few days and there is no necessity to use hour- or day-scale resolution to reduce the number of iterations, as in simulations of later stages of bone healing, which progresses through months [Borgiani et al. (43); Nasello et al. (62)]. In light of an eventual upscaling of the model to 3 dimensions, the spatial and temporal resolutions used in this study might be adapted after performing convergence analyses.

At the molecular level, the *in silico* model accurately simulates the transition from a pro-inflammatory to an anti-inflammatory environment, replicating the dynamic changes in the concentration of specialized inflammatory cytokines that occur during the initial phases of bone fracture healing [Maruyama et al. (13)]. Within the healing region, it is possible to observe a first pro-inflammatory wave of TNFα, with peak concentrations in the marginal regions during the first hours. This is followed by a progressive invasion of the defect site as the inflammatory response progresses (**Figure 3C**). The IL10 concentration was more prominent in the healing region around 2 days post-operation (**Figure 3D**), generating an anti-inflammatory environment to extinguish the inflammatory response and progress to the following repair stage. Modulation of the duration of the pro- and anti-inflammatory phases is critical to avoid unnecessary extended inflammation, which may lead to chronicity [Loi et al. (9)]. Therefore, the multiscale computer model might be used to investigate the two-way interactions between the cellular and molecular levels to predict how regulations at the smaller scale can have spatial-related implications on larger scales. Exogenous provision of treatments can be implemented at the molecular level by simulating a user-defined concentration spike in the healing region within a defined spatio-temporal frame. Molecular therapeutics targeting the inflammatory response, such as non-steroidal anti-inflammatory drugs [Lisowska et al. (63)], could be preliminarily tested with COMMBINI to investigate their effect on enhancing bone healing at the cellular level.

The computer model parametrized with literature data predicted a lower macrophage concentration within the callus region when compared to experimental data. To improve the model predictions, we performed a sensitivity analysis on the model outputs, followed by a sensitivity analysis on the model outputs followed by optimization of the most influential parameters using a GA and experimental results from a dedicated *in vivo* experiment. The sensitivity analysis showed that the model was particularly sensitive to changes in the macrophage recruitment ratio during the initial stage of healing and to the macrophage proliferation constant in the later inflammation. This result follows the expected monotonic relationship between the recruitment and proliferation ratio values and the macrophage concentration within the healing region. The GA calibration with experimental results on day 3 post-operation confirmed that an increasing value of the macrophage proliferation ratio was necessary to reduce the difference in macrophage concentration between the *in silico* and experimental results. The literature data (**Supplementary Tables 1, 2**), which we used to originally parametrize the model, underestimates the capacity of macrophages to proliferate within the healing region. Specifically, the value assigned to macrophage proliferation ratio has been obtained from *in vitro* cellular assays of isolated mature macrophages [Chitu et al. (64)]. However, while performing *in vitro* experiments on macrophages is less challenging than *in vivo*, only these last provide more exhaustive information on the behavior of those cells [Luque-Martin et al. (65)]. A valid compromise might be the use of advanced *in vitro* models, as organ-on-chip, to generate the investigative environment that more closely resembles the inflammatory scenario [Wikswo (66); Zhang et al. (67)]. Additionally, increasing the range of the GA (beyond the current upper bound of 50% variation) and including additional targets beyond the general macrophage concentration (*e.g.* macrophage subtypes) could further enhance the calibration.

To ensure accurate alignment between the simulated and experimental results, we developed an *in silico* immunofluorescence pipeline. In the simulation results, each macrophage subtype is visualized with a specific color, corresponding to the fluorescent staining used for the corresponding macrophages observed in the immunofluorescent images of the experimental outcomes. The calibration of the model was performed by quantitatively comparing the macrophage concentration inside a user-defined ROI on both *in silico* and experimental immunofluorescent images. The same procedure was employed to validate the model results with a second set of immunofluorescence images obtained from an independent experiment performed in murine femurs with a 0.7 mm osteotomy, collected at day 3 post-operation. The *in silico* fracture geometry was adapted to the validation dataset by reducing the dimension within the callus domain, while the model itself remained unaltered. Similar to the trend observed from experimental images, the *in silico* model predicted a reduced macrophage concentration for the smaller fracture gap. The validation data set was smaller than the calibration data set (*n* = 2) but we deemed it sufficient for the purpose of this proof of concept study where the focus is on the model development, calibration and the use of *in silico* immunofluorescence. In follow-up studies, when additional features will be added to the model (*e.g.* third spatial dimension, influence of mechanical loading), dedicated validation experiments will be run with sufficient power, including additional time points and spatial information to validate all aspects of the cellular and intracellular dynamics. Additionally, while the original parameter set used to calibrate the model was obtained from a male mouse population, the validation was performed in female animals. Macrophage characteristics in mice have been observed to be diverse between males and females [Chen et al. (68); Varghese et al. (69)]. Nevertheless, no obvious sex-specific influences were detected between the calibration and validation phase, though this might be due also to other potentially influencing factors such as age and strain. In this study, we have developed the model to capture normal healing in healthy adult mice. Its behavior when simulating other (patho)physiological states (ageing, disease-associated alterations or genetic modification), will be the subject of follow-up studies.

The model presented in this work aims to fill a wide gap in the *in silico* skeletal modeling field. While most of the state-of-the-art models limit their analysis to the later stages of bone fracture healing (repair and remodeling) [Ghiasi et al. (70); Borgiani et al. (35)], COMMBINI provides a new perspective on the role of the immune response in supporting and guiding bone healing during the first hours and days post-injury. The project’s overall aim is to build a mechano-biological environment that can simulate how changes at the molecular level (*e.g.* administration of exogenous pro-/anti-inflammatory cytokine) and the cellular level (*e.g.* specialized macrophage colonies seeded on a scaffold) might affect bone tissue regeneration. To date, COMMBINI includes only the biological regulators of the inflammatory phase. Future work will include the role of mechanical loading (*e.g.* from gait) on the regulation of the biological processes as it is well known that macrophages are mechanosensitive cells [Li et al. (71)]. The inclusion of the mechanical loading will add another source of (spatial) variation in the model, which might allow to capture the spatially non-uniform distribution in macrophage subtype observed experimentally [Stefanowski et al. (72)]. Additional limitations that will be included in future iterations of COMMBINI are the inclusion of cytokine chemotaxis [Edderkaoui (73)] and further refinement of the multiscale regulations of the macrophage population dynamics related to the development of the natural pro- and anti-inflammatory environment [Schlundt et al. (26); McCauley et al. (74); Frade et al. (75)]. Moreover, to limit the computational complexity of the current model, COMMBINI excludes the investigation of adaptive immune cells. Adaptive response plays a role in the late inflammatory stage and, therefore, its regulation is relevant for the subsequent regeneration stages [Baht et al. (10); Bucher et al. (76)]. The inclusion of additional macrophage subsets (*e.g.* M2 subsets: M2a, M2b, M2c, M2d) and lymphocytes could increase granularity at the cellular level and it is a possible route to cover also the subsequent repair and remodeling stages with this model [Bucher et al. (17); Gharavi et al. (77); Nikovics et al. (78)].

The model will be extended to include the transition into the early repair stage of bone healing, characterized by skeletal tissue formation. The addition of specialized cells (*e.g.* skeletal progenitor cells, osteoblasts, endothelial cells) will simulate the progression from the inflammatory to the repair stage and the revascularization within the healing region. Finally, the current simulation version of the model has been executed in 2D which is a choice made in relation to compute costs and the calibration/validation data available. In order to validate the 3-dimensional version of the model, 3D imaging techniques or reconstruction of stacked 2D slices will be required.

With the presented model, we developed a calibrated tool to investigate bone fracture healing progression starting from the initial inflammatory stage. To date, COMMBINI can simulate the natural innate immune response progression but will integrate the role of external interferences in the future. We believe that the *in silico* approach could favor a novel predictive strategy to plan adequate therapeutical strategies before surgical intervention when disruptive mechano-biological conditions occur (*e.g.* wide segmental defect, chronic inflammation). Furthermore, due to its multiscale nature, the model will be able to include alteration of the tissue, cell or molecular environment related to skeletal diseases. Osteomyelitis is a bacterial infection of the bone that might occur in case of open fracture [Slyamova et al. (79)]. The computational model can be integrated with the bacterial population and antibiotic treatment provision to investigate the role of the treatment on the infection and its influence on the natural development of the inflammatory response. The possibility of predicting the quantitative and qualitative outcome of the treatment strategy before its practical application will assist the operator in choosing the optimal path to follow, especially in case of challenging scenarios. For example, the impact of scaffolding the fracture with smart biomaterials, which sense environmental stimuli and respond accordingly, can be evaluated *in silico* with this model. The COMMBINI project fits well in the new trend of *in silico* trials [Pappalardo et al. (80); Viceconti et al. (81)] where validated computer models are employed to better inform or augment traditional *in vitro* and *in vivo* (animal and human) studies during the development of new therapeutic strategies.

## 5 Conclusions

With COMMBINI we developed a multiscale integrated *in silico* model for the study of the early inflammatory stage of bone fracture healing. An original approach with *in silico* immunofluorescence was presented and employed to calibrate the model with data from *in vivo* experiments. The calibration with a GA showed that *in vitro* models could not fully capture the macrophage proliferation process during bone healing inflammation. The validation with data from an independent experiment demonstrated the capacity of COMMBINI to capture the essential biological elements at play during the inflammatory phase of bone healing.

## Data availability statement

The raw data supporting the conclusions of this article will be made available by the authors upon request, without undue reservation.

## Author contributions

EB and LGe conceptualized and designed the study; EB and GN developed the computer model and the *in silico* immunofluorescence; GN, LO and TH designed the *in vivo* study that was used to calibrate the model; GN, LO, TH, and LGr performed the surgeries on the animals and collected the bone samples; EB and LO performed immunofluorescence on bone samples; EB developed the code to run the sensitivity analysis and Genetic Algorithm calibration; CHB and KS-B provided the experimental data for the validation set. All the authors helped with the analysis of the experimental results. EB wrote the first draft of the manuscript and all the other authors contributed to the article. All authors approved the submitted version.
