# Supplementary material for: COMMBINI: an experimentally-informed COmputational Model of Macrophage dynamics in the Bone INjury Immunoresponse
Source: Front Immunol. 2023 Nov 8;14:1231329. doi: 10.3389/fimmu.2023.1231329 (PMC10733790; doi:10.3389/fimmu.2023.1231329)
Supplement: Supplementary file 1 [file DataSheet_1.docx]

Supplementary Material

COMMBINI: an experimentally-informed
COmputational Model of Macrophage dynamics
in the Bone INjury Immunoresponse

**Edoardo Borgiani ^1,2,3,∗^, Gabriele Nasello ^2,4^, Liesbeth Ory ^2,4^, Tim Herpelinck ^2,4^, Lisanne Groeneveldt ^2,4,5^, Christian H. Bucher ^6^, Katharina Schmidt-Bleek ^6^ and Liesbet Geris ^1,2,3,4^**

^1^ Biomechanics Research Unit, GIGA In silico Medicine, University of Liège, Liège, Belgium
^2^ Prometheus, Division of Skeletal Tissue Engineering, KU Leuven, Leuven, Belgium
^3^ Division of Biomechanics, Department of Mechanical Engineering, KU Leuven, Leuven, Belgium
^4^ Skeletal Biology and Engineering Research Center, KU Leuven, Leuven, Belgium
^5^ Department of Cell Biology, Erasmus University Medical Center, Rotterdam, Netherlands
^6^ Julius Wolff Institute, Berlin Institute of Health, Charité - Universitätsmedizin Berlin, Berlin, Germany

*** Correspondence:** Edoardo Borgiani: [edoardo.borgiani@uliege.be](mailto:edoardo.borgiani@uliege.be)

# PARAMETER VALUES FROM LITERATURE

| Initial concentration within the healing region | $\left[ \boldsymbol{PMN} \right]_{\boldsymbol{0}}$ | PMN | 1.00 10^-4^ µm^-3^ | Ass. |
| --- | --- | --- | --- | --- |
| Maximum recruitment factor from surrounding tissues | $\boldsymbol{k}_{\boldsymbol{R}}$ | M0, PMN | 2.10 10^-2^ h^-1^ | Ass. |
| Maximum cellular concentration | $\boldsymbol{[}{\boldsymbol{M}\boldsymbol{\Phi}\boldsymbol{]}}_{\boldsymbol{max}}$ | MΦ | 1.00 10^-4^ µm^-3^ | Ass. |
|  | $\boldsymbol{[PMN]}_{\boldsymbol{max}}$ | PMN | 1.00 10^-3^ µm^-3^ | Ass. |
| Cellular migration speed | $\boldsymbol{k}_{\boldsymbol{v}}$ | MΦ | 1.00 µm min^-1^ | [a,b] |
|  |  | PMN | 5.00 µm min^-1^ | [c] |
| Cellular proliferation/doubling ratio | $\boldsymbol{k}_{\boldsymbol{p}}$ | MΦ | 8.33 10^-4^ min^-1^ | [d] |
| Cellular apoptosis ratio | $\boldsymbol{k}_{\boldsymbol{a}}$ | MΦ | 2.36 10^-5^ min^-1^ | [e] |
|  |  | PMN | 7.00 10^-5^ min^-1^ | [f] |
| Debris engulfment ratio | $\boldsymbol{k}_{\boldsymbol{e}}$ | MΦ | 3.33 10^-2^ min^-1^ | [g] |
|  |  | PMN | 3.33 10^-3^ min^-1^ | Ass. |
| Macrophage polarization ratio | $\boldsymbol{k}_{\boldsymbol{01}}$ | M0 → M1 | 4.17 10^-4^ min^-1^ | [h] |
|  | $\boldsymbol{k}_{\boldsymbol{02}}$ | M0 → M2 | 2.08 10^-4^ min^-1^ | [h] |
|  | $\boldsymbol{k}_{\boldsymbol{12}}$ | M1 → M2 | 5.76 10^-5^ min^-1^ | [h] |
|  | $\boldsymbol{k}_{\boldsymbol{21}}$ | M2 → M1 | 3.47 10^-6^ min^-1^ | [h] |
| Cytokine half-saturation constant in macrophage polarization | $\boldsymbol{a}_{\boldsymbol{01}}$ | M0 → M1  (TNFα) | 1.00 10^-13^ ng µm^-3^ | [h] |
|  | $\boldsymbol{a}_{\boldsymbol{02}}$ | M0 → M2 (IL10) | 5.00 10^-13^ ng µm^-3^ | [h] |
|  | $\boldsymbol{a}_{\boldsymbol{12}}$ | M1 → M2 (IL10) | 1.00 10^-13^ ng µm^-3^ | [h] |
|  | $\boldsymbol{a}_{\boldsymbol{21}}$ | M2 → M1 (TNFα) | 5.00 10^-13^ ng µm^-3^ | [h] |
| TNFα secretion ratio | $\boldsymbol{k}_{\boldsymbol{TNF}}$ | M0, M1 | 5.78 10^-9^ ng cell^-1^ min^-1^ | [j] |
|  |  | PMN | 4.61 10^-10^ ng cell^-1^ min^-1^ | [k] |
| IL10 secretion ratio | $\boldsymbol{k}_{\boldsymbol{IL}\boldsymbol{10}}$ | M0 | 1.27 10^-9^ ng cell^-1^ min^-1^ | [j] |
|  |  | M2 | 2.58 10^-9^ ng cell^-1^ min^-1^ | [j] |
| TGFβ secretion ratio | $\boldsymbol{k}_{\boldsymbol{TGF}}$ | M0, M1 | 3.13 10^-8^ ng cell^-1^ min^-1^ | [l] |
|  |  | M2 | 2.67 10^-10^ ng cell^-1^ min^-1^ | [l] |
| IFNγ secretion ratio | $\boldsymbol{k}_{\boldsymbol{IFN}}$ | M0, M1 | 5.20 10^-12^ ng cell^-1^ min^-1^ | [m] |
|  |  | PMN | 2.06 10^-10^ ng cell^-1^ min^-1^ | [n] |

**Supplementary Table 1.** Literature and assumed (Ass.) values used to parametric define the cellular level in the biological module of COMMBINI. MΦ: macrophages, M0: non-polarized macrophages, M1: pro-inflammatory macrophages, M2: anti-inflammatory macrophages, PMN: polymorphonuclear neutrophils, TNFα: tumor necrosis factor alpha, IL10: interleukin-10, TGFβ: Transforming Growth Factor beta, IFNγ: Interferon gamma.

a:Segovia-Juarez et al. (2004), b:Pixley (2012), c:Hoang et al. (2013), d:Chitu et al. (2011), e:Catelas et al. (2005), f:Kovtun et al. (2018), g:Nagaraja et al. (2014), h:Trejo et al. (2019), j:Byrne and Reen (2002), k:Cassatella (1995), l:Fadok et al. (1998), m:Munder et al. (1998), n:Ethuin et al. (2004).

| Initial concentration within the healing region | Debris | $\boldsymbol{Db}_{\mathbf{0}}$ | 1.00 10^-4^ ng mL^-1^ | Ass. |
| --- | --- | --- | --- | --- |
| TNFα secretion suppression equation parameters | IL10 | $\boldsymbol{k}_{\boldsymbol{TNIL}}$ | 4.60 10^-1^ | [a] |
|  |  | $\boldsymbol{a}_{\boldsymbol{TNIL}}$ | 1.53 10^12^ µm^3^ ng^-1^ | [a] |
|  |  | $\boldsymbol{b}_{\boldsymbol{TNIL}}$ | 5.44 10^-1^ | [a] |
|  | TGFβ | $\boldsymbol{k}_{\boldsymbol{TNTG}}$ | 6.21 10^-1^ | [a] |
|  |  | $\boldsymbol{a}_{\boldsymbol{TNTG}}$ | 8.30 10^11^ µm^3^ ng^-1^ | [a] |
|  |  | $\boldsymbol{b}_{\boldsymbol{TNTG}}$ | 4.47 10^-1^ | [a] |
|  | IFNγ | $\boldsymbol{k}_{\boldsymbol{TNI}}$ | 2.00 10^-1^ | [b] |
|  |  | $\boldsymbol{a}_{\boldsymbol{TNI}}$ | 6.00 10^-9^ ng µm^-3^ | [b] |
| IFNγ secretion suppression equation parameters | TNFα | $\boldsymbol{a}_{\boldsymbol{ITN}}$ | 1.64 10^10^ µm^3^ ng^-1^ | Ass. |
| Cytokine diffusion coefficient | $\boldsymbol{D}$ | Debris | 1.00 µm^2^ min^-1^ | Ass. |
|  |  | TNFα | 1.80 10^3^ µm^2^ min^-1^ | # |
|  |  | IL10 | 1.80 10^3^ µm^2^ min^-1^ | # |
|  |  | TGFβ | 1.56 10^3^ µm^2^ min^-1^ | # |
|  |  | IFNγ | 1.70 10^3^ µm^2^ min^-1^ | # |
| Cytokine decay rate | $\boldsymbol{d}$ | TNFα | 8.90 10^-3^ min^-1^ | [c] |
|  |  | IL10 | 3.22 10^-3^ min^-1^ | [d] |
|  |  | TGFβ | 1.16 10^-2^ min^-1^ | [e] |
|  |  | IFNγ | 3.22 10^-3^ min^-1^ | Ass. |

**Supplementary Table 2.** Literature and assumed (Ass.) values used to parametric define the molecular level in the biological module of COMMBINI. TNFα: tumor necrosis factor alpha, IL10: interleukin-10, TGFβ: Transforming Growth Factor beta, IFNγ: Interferon gamma.

a:Nagaraja et al. (2014), b:Vila-del Sol et al. (2008), c:Wong et al. (2001), d:Reynolds et al. (2006), e:Werner and Grose (2003), #: calculated as the inverse of the cube root of the molecular weight (*https://personal.math.ubc.ca/ ais/website/status/diffuse.html*).

# SENSITIVITY ANALYSIS RESULTS

|  |  | **Day 1** | **Day 3** |
| --- | --- | --- | --- |
| $\left[ \boldsymbol{PMN} \right]_{\boldsymbol{0}}$ | PMN | -11.70% | -37.49% |
| $\boldsymbol{k}_{\boldsymbol{R}}$ | M0 | 46.96% | 3.33% |
| $\boldsymbol{k}_{\boldsymbol{p}}$ | M0 | 12.93% | 4.78% |
|  | M1 | 1.98% | 15.94% |
|  | M2 | 0.10% | 3.15% |
| $\boldsymbol{k}_{\boldsymbol{a}}$ | M0 | <0.01% | -0.08% |
|  | M1 | <0.01% | <0.01% |
|  | M2 | -0.04% | <0.01% |
| $\boldsymbol{k}_{\boldsymbol{e}}$ | PMN | -7.62% | -13.37% |
|  | MΦ | -9.53% | -1.60% |
| $\boldsymbol{k}_{\boldsymbol{01}}$ | M0 → M1 | <0.01% | 0.28% |
| $\boldsymbol{k}_{\boldsymbol{02}}$ | M0 → M2 | 0.10% | 0.09% |
| $\boldsymbol{k}_{\boldsymbol{12}}$ | M1 → M2 | 0.02% | <0.01% |
| $\boldsymbol{k}_{\boldsymbol{21}}$ | M2 → M1 | -0.01% | -0.02% |
| $\boldsymbol{a}_{\boldsymbol{01}}$ | M0 → M1 | 0.02% | 0.03% |
| $\boldsymbol{a}_{\boldsymbol{02}}$ | M0 → M2 | <0.01% | <0.01% |
| $\boldsymbol{a}_{\boldsymbol{12}}$ | M1 → M2 | <0.01% | -0.05% |
| $\boldsymbol{a}_{\boldsymbol{21}}$ | M2 → M1 | <0.01% | <0.01% |
| $\boldsymbol{k}_{\boldsymbol{TNF}}$ | M0 | -0.01% | 0.06% |
|  | M1 | -0.06% | -0.17% |
| $\boldsymbol{k}_{\boldsymbol{IL}\boldsymbol{10}}$ | M0 | <0.01% | 0.32% |
|  | M2 | <0.01% | 0.01% |
| $\boldsymbol{k}_{\boldsymbol{TGF}}$ | M0 | <0.01% | <0.01% |
|  | M1 | <0.01% | -0.05% |
|  | M2 | -0.01% | 0.47% |
| $\boldsymbol{k}_{\boldsymbol{IFN}}$ | M0 | -0.06% | 0.06% |
|  | M1 | 0.09% | 0.03% |
|  | PMN | <0.01% | -0.02% |
| $\boldsymbol{k}_{\boldsymbol{TNIL}}$ | IL10 → TNFα | 0.01% | -0.02% |
| $\boldsymbol{k}_{\boldsymbol{TNTG}}$ | TGFβ → TNFα | <0.01% | -0.09% |
| $\boldsymbol{k}_{\boldsymbol{TNI}}$ | IFNγ → TNFα | -0.03% | <0.01% |
| $\boldsymbol{D}$ | Debris | 4.43% | 7.47% |
|  | TNFα | -0.13% | -0.18% |
|  | IL10 | <0.01% | -0.06% |
| $\boldsymbol{d}$ | TNFα | <0.01% | -0.04% |
|  | IL10 | <0.01% | 0.05% |

**Supplementary Table 3.** Percentage of the total sum of squares calculated with Analysis of Variance to evaluate the sensitivity of the model to its parameters when macrophage concentration was predicted at day 1 and 3 post-fracture. MΦ: macrophages, M0: non-polarized macrophages, M1: pro-inflammatory macrophages, M2: anti-inflammatory macrophages, PMN: polymorphonuclear neutrophils, TNFα: tumor necrosis factor alpha, IL10: interleukin-10, TGFβ: Transforming Growth Factor beta, IFNγ: Interferon gamma.

# GENETIC ALGORITHM

The calibration of the computer model has been performed using a Genetic Algortihm (GA) (McCall (2005)). A multi-parameter approach has been employed to perform a global optimization of the model. Multiple variables have been optimized in parallel to find the best combination of values that can lead to a computational output more similar to the experimental observations. The GA is an evolutionary optimization scheme, achieving its goal through subsequent optimization steps that aim to minimize a fitness function. In this case, the cellular concentration difference between *in silico* and experimental immunofluorescence, in absolute value, has been chosen as the fitness function. The GA described here is user-regulated by defining hyperparameters ($\boldsymbol{N}_{\boldsymbol{P}}$, $\boldsymbol{N}_{\boldsymbol{B}}$, $\boldsymbol{N}_{\boldsymbol{Pop}}$, $\boldsymbol{P}_{\boldsymbol{C}}$, $\boldsymbol{P}_{\boldsymbol{M}}$), which can be adapted to improve the performance and efficacy of the optimization process. The optimization has been performed on the $\boldsymbol{N}_{\boldsymbol{P}}$ most influential parameters, identified by Analysis of Variance on the results from an initial screening experiment using Design of Experiments (§ 2.6 in the main manuscript). The initial value of those parameters has been taken from literature data. The optimization of each value spans between a range of +/- 50% of the initial value. While the original GA method allows an investigation between two levels (+50% or -50%), we analyzed a wide spectrum by using a $\boldsymbol{N}_{\boldsymbol{B}}$-bit binary codification (Supp. Figure 1). This methodology extended the investigation by including all the values in between with a user-defined number of equidistant steps ($2^{\boldsymbol{N}_{\boldsymbol{B}}}$). The GA generated a population of $\boldsymbol{N}_{\boldsymbol{Pop}}$ samples, where each of the investigated parameters is randomly assigned to one value within the range. Each sample is identified by a binary string of $\boldsymbol{N}_{\boldsymbol{P}}$ ×$\boldsymbol{N}_{\boldsymbol{B}}$ figures that identify the parameter values chosen for that sample. To initialize the process, a value is assigned to each parameter by randomly appointing 0 or 1 to each figure of each sample, therefore creating a heterogeneous population to investigate (Supp. Figure 1). The stochastic population that is created in this way characterizes the initial population (Generation 0).


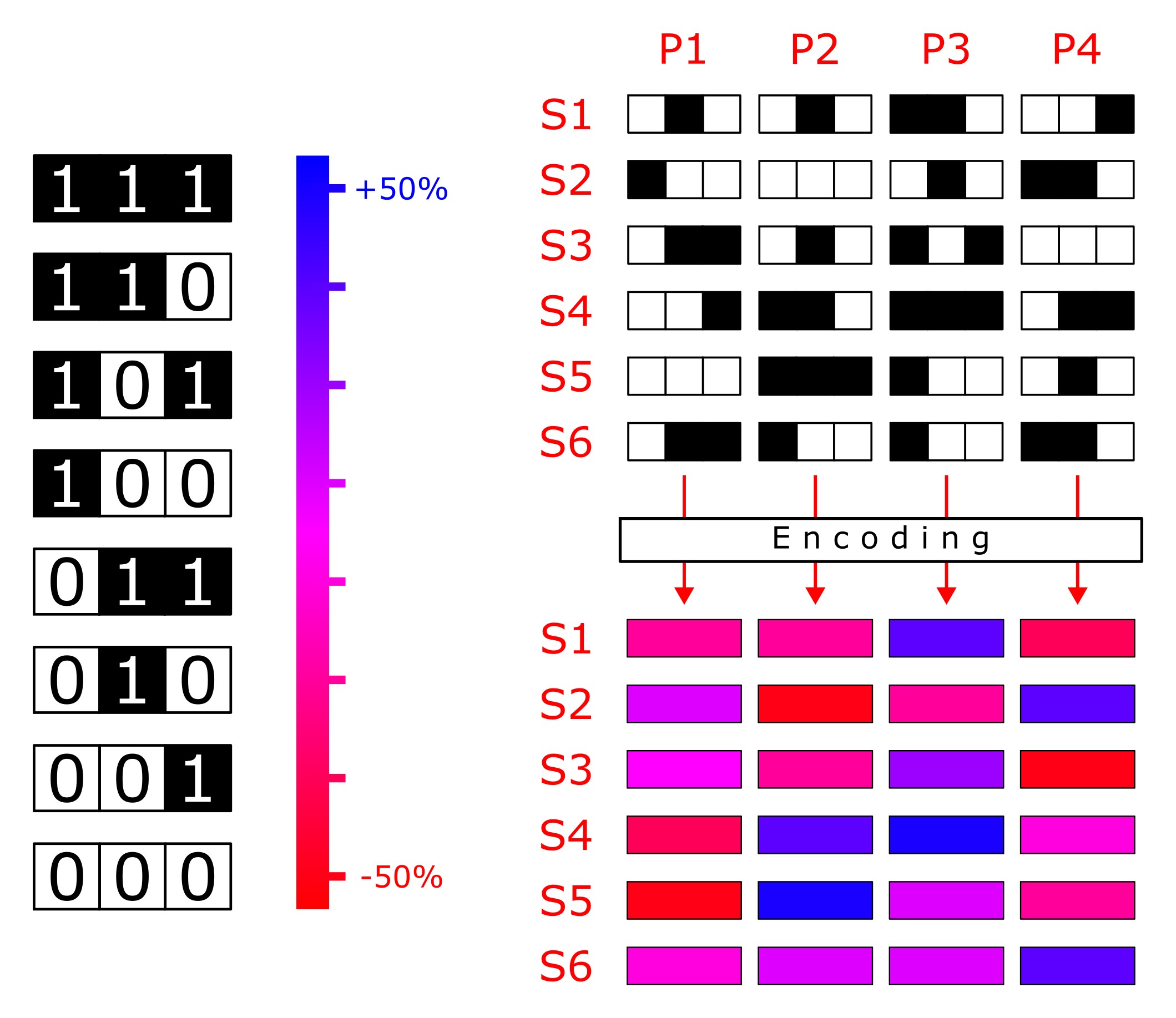


**Supplementary Figure 1.** Random creation of Generation 0 population associated to the encoding algorithm (left). In this example $\boldsymbol{N}_{\boldsymbol{P}}$=4 (4 parameters, P1 – P4), $\boldsymbol{N}_{\boldsymbol{Pop}}$=6 (6 samples in each population, S1 – S6), $\boldsymbol{N}_{\boldsymbol{B}}$=3 (3-bit encoded algorithm, 2^3^ values). The first population is generated by randomly assigning 0 and 1 to each binary digit and encoding each parameter within a value in the ± 50% range.

The model runs once per each sample with a specific combination of parameters and the output is quantitatively compared with the experimental immunofluorescent images. The output difference is the fitness function that we aim to minimize with the GA. The evolutionary optimization progresses by following a generation-based adaptation of the parametric values to reduce the fitness function. The members of each population are subject to a “tournament selection” to decide the ones that deserve to pass to the following generation. This selection method is based on a 1-vs-1 competition where each sample is compared with a sample within the same generation, chosen randomly. The “winner” sample is the one with the smaller quantitative difference from the experimental data, therefore it is the one that contributes the most to reduce our fitness function. It does not enter directly into the population group in the following generation but it is listed in a “parents” group, that contains the samples that deserve to pass to the next generation, due to their higher contribution in reducing the fitness function (Supp. Figure 2).


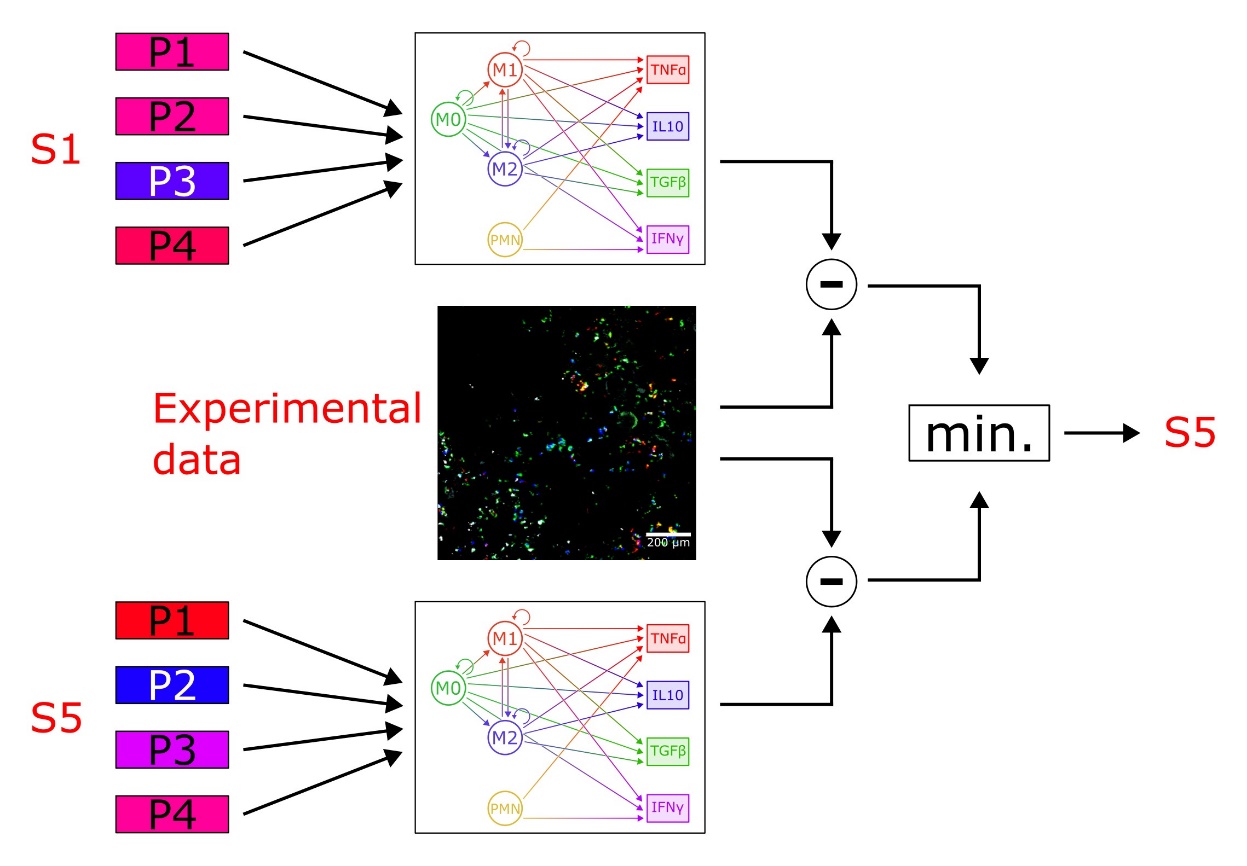


**Supplementary Figure 2.** “Tournament selection” algorithm between sample S1 and a second sample (chosen randomly, sample S5 in this example). The quantitative difference between the model output, when sample-specific parameter values are used, and the experimental data are compared. The sample that provides the smallest difference passes to the “parents” list (S5 in this case). This process is iteratively performed for all samples.

Those “parents” are randomly coupled to generate the population of the next generation. A crossover probability value ($\boldsymbol{P}_{\boldsymbol{C}}$) has been imposed to define the probability that a parameter crossover happens between the samples in the couples. The crossover between a couple of “parents” is the feature that provides the name “genetic” to the GA. There is an exchange of parameters (as “genes”) between the two parents in the creation of two of the samples of the next generation (Supp. Figure 3). The exchange involves a number between 1 to $\boldsymbol{N}_{\boldsymbol{P}}$ -1 parameters, chosen randomly. In addition, a mutation probability ($\boldsymbol{P}_{\boldsymbol{M}}$) has been included in the model to extend the investigation range beyond the legacy of the “parents”. The mutation probability is the chance that one of the binary digits of the samples in the population is switched into the other digit (0→ 1 or 1→ 0).


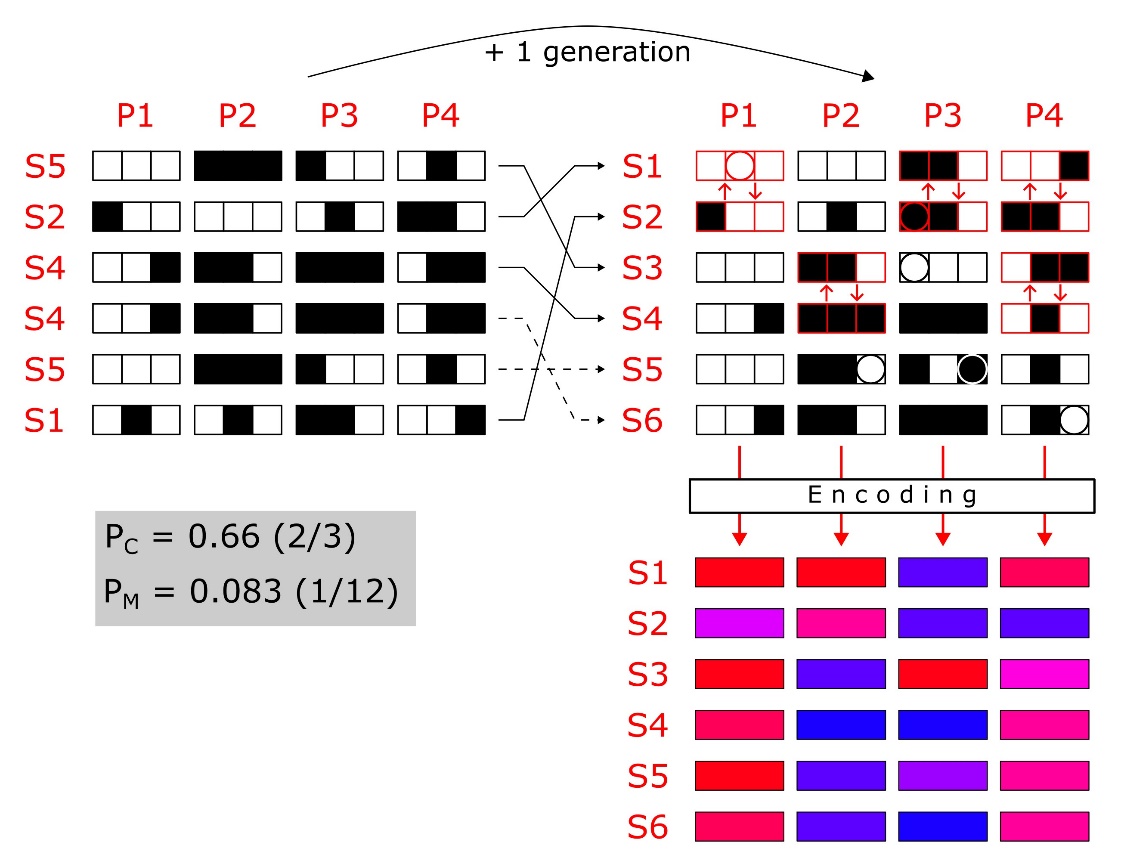


**Supplementary Figure 3.** The new generation (right) is generated by randomly coupling the “parents” (left). According to the $\boldsymbol{P}_{\boldsymbol{C}}$ used in this example, only two over three couples are subject to the crossover of some parameters. The crossovers, indicated by red frames and switching arrows, are performed by exchanging a randomly selected set of digits between the couples. Each couple that is not subject to crossover directly moves on to the mutation step (dashed arrows). The mutations are indicated by circles and consequent digit switches (here one every 12 digits, according to $\boldsymbol{P}_{\boldsymbol{M}}$).

The algorithm is iterated through multiple generations until the average value associated with the fitness function is subject to a transgenerational variation below a threshold of 5%. The sample in the last generation that provides the smallest quantitative difference between computational results and experiments is chosen as optimal to describe the parameter values. For the model presented in the manuscript, the hyperparameter values were $\boldsymbol{N}_{\boldsymbol{P}}$ = 4, $\boldsymbol{N}_{\boldsymbol{B}}$ = 6, $\boldsymbol{N}_{\boldsymbol{Pop}}$= 16, $\boldsymbol{P}_{\boldsymbol{C}}$= 0.9, $\boldsymbol{P}_{\boldsymbol{M}}$ = 0.04.

# REFERENCES

Byrne, A. and Reen, D. J. (2002). Lipopolysaccharide induces rapid production of IL-10 by monocytes in the presence of apoptotic neutrophils. *The Journal of Immunology* 168, 1968–1977. doi:10.4049/jimmunol.168.4.1968

Cassatella, M. A. (1995). The production of cytokines by polymorphonuclear neutrophils. *Immunology Today* 16, 21–26. doi:10.1016/0167-5699(95)80066-2

Catelas, I., Petit, A., Vali, H., Fragiskatos, C., Meilleur, R., Zukor, D. J., et al. (2005). Quantitative analysis of macrophage apoptosis vs. necrosis induced by cobalt and chromium ions in vitro. *Biomaterials* 26, 2441–2453. doi:10.1016/j.biomaterials.2004.08.004

Chitu, V., Yeung, Y., Yu, W., Nandi, S., and Stanley, E. R. (2011). Measurement of macrophage growth and differentiation. *Current Protocols in Immunology* 92. doi:10.1002/0471142735.im1420s92

Ethuin, F., Gérard, B., Benna, J. E., Boutten, A., Gougereot-Pocidalo, M.-A., Jacob, L., et al. (2004). Human neutrophils produce interferon gamma upon stimulation by interleukin-12. *Laboratory Investigation* 84, 1363–1371. doi:10.1038/labinvest.3700148

Fadok, V. A., Bratton, D. L., Konowal, A., Freed, P. W., Westcott, J. Y., and Henson, P. M. (1998). Macrophages that have ingested apoptotic cells in vitro inhibit proinflammatory cytokine production through autocrine/paracrine mechanisms involving TGF-beta, PGE2, and PAF. *Journal of Clinical Investigation* 101, 890–898. doi:10.1172/jci1112

Hoang, A. N., Jones, C. N., Dimisko, L., Hamza, B., Martel, J., Kojic, N., et al. (2013). Measuring neutrophil speed and directionality during chemotaxis, directly from a droplet of whole blood. *TECHNOLOGY* 01, 49–57. doi:10.1142/s2339547813500040

Kovtun, A., Messerer, D. A., Scharffetter-Kochanek, K., Huber-Lang, M., and Ignatius, A. (2018). Neutrophils in tissue trauma of the skin, bone, and lung: Two sides of the same coin. *Journal of Immunology Research* 2018, 1–12. doi:10.1155/2018/8173983

McCall, J. (2005). Genetic algorithms for modelling and optimisation. *Journal of Computational and Applied Mathematics* 184, 205–222. doi:https://doi.org/10.1016/j.cam.2004.07.034. Special Issue on Mathematics Applied to Immunology

Munder, M., Mallo, M., Eichmann, K., and Modolell, M. (1998). Murine macrophages secrete interferon γ upon combined stimulation with interleukin (IL)-12 and IL-18: A novel pathway of autocrine macrophage activation. *Journal of Experimental Medicine* 187, 2103–2108. doi:10.1084/jem.187.12.2103

Nagaraja, S., Wallqvist, A., Reifman, J., and Mitrophanov, A. Y. (2014). Computational approach to characterize causative factors and molecular indicators of chronic wound inflammation. *The Journal of Immunology* 192, 1824–1834. doi:10.4049/jimmunol.1302481

Pixley, F. J. (2012). Macrophage migration and its regulation by CSF-1. *International Journal of Cell Biology* 2012, 1–12. doi:10.1155/2012/501962

Reynolds, A., Rubin, J., Clermont, G., Day, J., Vodovotz, Y., and Bard Ermentrout, G. (2006). A reduced mathematical model of the acute inflammatory response: I. derivation of model and analysis of anti-inflammation. *Journal of Theoretical Biology* 242, 220–236. doi:10.1016/j.jtbi.2006.02.016

Segovia-Juarez, J. L., Ganguli, S., and Kirschner, D. (2004). Identifying control mechanisms of granuloma formation during m. tuberculosis infection using an agent-based model. *Journal of Theoretical Biology* 231, 357–376. doi:10.1016/j.jtbi.2004.06.031

Trejo, I., Kojouharov, H., and Chen-Charpentier, B. (2019). Modeling the macrophage-mediated inflammation involved in the bone fracture healing process. *Mathematical and Computational Applications* 24, 12. doi:10.3390/mca24010012

Vila-del Sol, V., Carmen, P., and Fresno, M. (2008). IFN-γ-induced TNF-α expression is regulated by interferon regulatory factors 1 and 8 in mouse macrophages. *The Journal of Immunology* 181, 4461–4470. doi:10.4049/jimmunol.181.7.4461

Werner, S. and Grose, R. (2003). Regulation of wound healing by growth factors and cytokines. *Physiological Reviews* 83, 835–870. doi:10.1152/physrev.2003.83.3.835

Wong, S.-S., Schwartz, R. C., and Pestka, J. J. (2001). Superinduction of TNF-α and IL-6 in macrophages by vomitoxin (deoxynivalenol) modulated by mRNA stabilization. *Toxicology* 161, 139–149. doi:10.1016/s0300-483x(01)00331-6
